# Human papillomavirus types in cases of squamous cell carcinoma of head and neck in Colombia

**DOI:** 10.5935/1808-8694.20130065

**Published:** 2015-10-04

**Authors:** Katherine Quintero, Gabriel A. Giraldo, Mary L. Uribe, Armando Baena, Carolina Lopez, Efrain Alvarez, Gloria I Sanchez

**Affiliations:** a MD (Young researcher - U of A, Infection and Cancer Group - Medical School - University of Antioquia, UdeA, Medellin, Colombia).; b DDS, MSC, Maxillofacial surgeon (Assistant Professor, Dentistry School - University of Antioquia and Las Vegas Clinic, Medellin, Colombia).; c BSc, MSc (PhD student, University of Alicante, Alicante, Spain).; d Ing., BSc statistician (PhD student of epidemiology, Infection and Cancer Group - University of Antioquia, UdeA, Medellin, Colombia).; e MD, Pathologist (Assistant professor, Department of Pathology - Medical School - University of Antioquia, Medellin, Colombia).; f DDS; MSc (Associate professor - School of Dentistry - University of Antioquia, Medellin, Colombia).; g MSc.; Ph.D (Associate Professor, Coordinator of the Infection and Cancer Group, University of Antioquia, UdeA, Medellin, Colombia). Infection and Cancer Group & Department of Pathology - School of Medicine; School of Dentistry; University of Antioquia, Medellin, Colombia.

**Keywords:** papillomavirus vaccines, oropharyngeal neoplasms, head and neck neoplasms, papillomavirus infections, laryngeal neoplasms

## Abstract

Estimating the type-specific prevalence of human papillomavirus (HPV) in head and neck cancer (HNSCC) is helpful in predicting the impact of HPV immunization.

**Objective:**

To estimate the overall prevalence, and gender and age-specific prevalence of HPV in HNSCC.

**Method:**

This cross sectional retrospective study was carried out in four pathology laboratories of Medellin, Colombia. HPV testing was performed by GP5+/6+ PCR-based RLB and HPV 16 and 18 type-specific PCR.

**Results:**

175 primary HNSCC cases consecutively diagnosed between 1999 and 2008 with confirmed diagnosis and amplifiable DNA were included. Overall HPV prevalence was 18.9%. HPV was found in 23.9%, 17.5% and 13.3% of the oral cavity, larynx and oropharynx cases respectively. Among HPV positive cases, 82% were HPV 16 and 18% were HPV 18. No other HPV genotypes were identified. Most patients were males. Male patients were younger that their female counterparts, particularly in oral cavity cancer cases.

**Conclusion:**

HPV 16 and 18 genotypes were found in nearly 20% of HNSCC cases in Colombian patients. The impact of HPV vaccination for the prevention of HNSCC in this population deserves further evaluation.

## INTRODUCTION

The head and neck squamous cell carcinoma (HNSCC) is the fifth most common cancer worldwide, with around 600.000 cases diagnosed in 2008^1^. Most of these cancers arise from squamous cells in the oral cavity, oropharynx, nasopharynx, hypopharynx and larynx. Smoking and alcohol drinking are known risk factors for these cancers^2^, and Epstein-Barr virus has been recognized as a cause of nasopharyngeal cancer^3^.

The relationship of Human Papillomavirus (HPV) with a subset of HNSCC cases has been firmly established^4^, ^5^, ^6^, ^7^. HPV positive HNSCC cases are associated with younger age, male predominance and risky sexual behaviors such as a high number of sexual partners and history of oral sex^8^, ^9^, ^10^. In contrast HPV-negative HNSCC is most likely related to cumulative measures of smoking and alcohol drinking and poor oral hygiene^11^. Cancers of the tonsils and base of the tongue are the sites more frequently associated with HPV but HPV has been also detected in oral cavity and larynx.

A systematic review that included 5,046 specimens of HNSCC of 60 studies that used PCR-based methods to detect the genotype of HPV^12^ showed and estimated prevalence of HPV DNA of about 25.9% of these tumors. The prevalence was significantly higher in cancer of the oropharynx (35.6%, range 11-100%) than in the oral cavity (23.5%, range 40% to 80%) or larynx (24.0%, range 0-100%) with HPV 16 being the predominant genotype found. Among the HPV-positive tumors, HPV 16 is the most frequent genotype in oropharyngeal HNSCC (86.7%) followed by larynx (69.2%) and oral (68.2%). HPV 18 is the second most frequent genotype detected: 2.8% in the oropharynx, 34.1% in the oral cavity and 17% in carcinomas of the larynx.

In 2009, the Internationl Agency for Research on Cancer evaluated the available evidence on the carcinogenicity of HPV in humans and concluded that there is sufficient evidence for the carcinogenicity of HPV 16 in the oral cavity, oropharynx, and tonsils and still limited evidence of HPV as a carcinogen in the larynx^3^. There are significant geographical and temporal variations in the incidence of HNSCC cancer. Although in Europe and North America, non-HPV related HNSCC has been declining, a time-trend increase in the incidence of HPV-related oropharyngeal cancer has been observed in the last decade^9^, ^13^, ^14^, ^15^.

It has been suggested that regional variations in global and site-specific incidence of HNSCC seem to relate to alcohol and tobacco use and that temporal variations may be due to changes in sexual behavior and increased exposure to HPV infection^16^, ^17^. In spite of a very low HPV prevalence in HNSCC cases^18^, Brazil has the highest incidence rate of HNSCC among South American countries^1^, ^16^. Prevention of diseases caused by HPV underlies on the introduction of prophylactic vaccines which are highly effective in preventing HPV infection^19^, ^20^, ^21^. Currently available vaccines include HPV 16 and 18, the 2 most prevalent genotypes in HNSCC. Therefore, it is expected that these vaccines have an impact on this type of cancers^22^.

There are not many studies in South America evaluating the prevalence of HPV in HNSCC. The aim of this study was to determine the prevalence of HPV genotypes by age and gender in cancer of the oral cavity, oropharynx and larynx, in four laboratories of pathology from the city of Medellin in Colombia.

## METHOD

### Study Design and Population

This is a cross-sectional retrospective survey. One hundred and seventy-five cases of HNSCC, diagnosed consecutively between 1999 and 2008, identified in the records of the departments of pathology at the medical and dental schools of the University of Antioquia, and in the laboratory of pathology and cytology and the department of pathology of the University Hospital Fundacion San Vicente de Paul were included in the study. The histological slides of each case were collected for confirmation of pathological diagnosis and the respective paraffin blocks of the confirmed cases retrieved for further analysis. Epidemiological information on sex, age, year of diagnosis and location of the lesion were obtained from the pathology reports.

### Microdissection and DNA Extraction

Five histological sections were obtained from each paraffin block, wherein the first and the last sections stained with hematoxylin & eosin (H & E) were used for histological evaluation and the remaining sections for microdissection of the lesion. After histological confirmation, lesions were circled and corresponding tumor areas microdissected from unstained slides using a sterile surgical scalpel in each case. The dissected material was transferred to non-silicone tubes and xylene (350 µL) was added to each sample to dissolve the paraffin. Paraffin-free tissue was precipitated with 150 µL of cold 100% ethanol and centrifuged and the pellet was allowed to dry at room temperature overnight. Dry button was resuspended in 100 µL of proteinase K buffer (10 mg/mL proteinase K in 50 mM Tris, pH 8.3) and incubated overnight at 37°C. Finally the samples were incubated at 95°C for 8 min to inactivate proteinase K and stored at -20°C until use. Paraffin blocks sectioning and DNA extraction was conducted under strict conditions to avoid contamination. Blank paraffin blocks with normal tissue were included among every 22 samples processed, and positive controls were paraffin-embedded cervical cancer tissues positive for HPV. DNA quality was evaluated by amplifying a 209 bp segment of the human β-globin gene.

### HPV DNA detection

Initially, HPV DNA was detected by the HPV general primer GP5+/6+ mediated PCR, as described previously^23^ and followed by genotyping of PCR products with reverseline blot hybridization with specific probes for 37 different HPV genotypes (HPV types 6, 11, 16, 18, 26, 31, 33, 34, 35, 39, 40, 42, 43, 44, 45, 51, 52, 53, 54, 55, 56, 57, 58, 59, 61, 66, 68, 70, 71, 72, 73, 81, 82/IS39, 82/MM4, 83, 84, CP6108). All samples negative for hybridization after the GP5+/GP6+ mediated PCR, were subjected to HPV 16 and HPV 18 genotype specific PCR as described^24^. The primers used in this procedure amplify a fragment of 96 pb (HPV-16) and 115 pb (HPV-18) of the E6 gene, which was visualized in 2% agarose gels stained with ethidium bromide.

### Statistical analysis

The cases were described by age, sex and tumor location. The percentage of positive cases for any HPV genotype, HPV 16 and HPV 18, was estimated among good quality samples (β-globin positive or/and HPV positive). A descriptive analysis of the frequency of the epidemiological characteristics of cases and HPV infection was conducted. Subsequently, the risk of HPV infection (for any genotype) was estimated by crude or adjusted by age odds ratios, with their respective confidence intervals using logistic regression. The nonparametric Mann-Whitney test was used to compare differences in age according to HPV status.

These tests were also performed for HPV-16, the most common genotype. A significance level of 0.05 was used for all analysis and results were generated using the statistical package R version 2.12.2 (R Development Core Team (2011). R: A language and environment for statistical computing. R Foundation for Statistical Computing, Vienna, Austria. ISBN 3-900051-07-0, URL. (http://www.R-project.org/http://www.R-project.org/).

### Ethical considerations

The study was approved by the bioethics committee of the University of Antioquia Research Committee of the school of dentistry (Concept 02-2011, May 18, 2011). Privacy, confidentiality and anonymity were assured in all procedures and the World Medical Association Declaration of Helsinki as well as the ethical principles for medical research involving human subjects contained in the International Ethical Guidelines for Epidemiological Studies prepared by the Council for International Organizations of Medical Sciences (CIOMS) in collaboration with the World Health Organization (WHO) for 2008, were closely followed.

## RESULTS

### Description of the cases

The characteristics of the 175 cases included in the analysis are described in Table 1. Thirty-eight point three percent, 36% and 25.7% of lesions were from oral cavity, larynx and oropharynx respectively. Age of cases ranged from 22 to 97 years and the mean age/(standard deviation) at diagnosis was 64.5/(12.1) years. Overall, there was no difference in the mean age/SD at diagnosis among lesions from different sites but cases of oral cavity were diagnosed at a younger age in men (data not shown). The vast majority of cases were between 50 to 79 years old and males (63.4%, 111/175). The distribution of cases in the oral cavity was similar between males and females. However, the percentage of cases from larynx (20.6% vs. 79.4%) and oropharynx (37.8% vs. 62.2%) was higher in males than in females and this was a statistically significant difference (Chi-Square test *p* = 0.0017).

**Table 1:** Characteristics of cases of Head and Neck and Cancer of clinical centers of Medellin, Colombia.

					Anatomic sites				
Characteristics	Total		Oral cavity		Larynx		Oropharynx		*p*^a^
	n	%	n	%	n	%	n	%	
Total	175	100.0	67	38.3	63	36	45	25.7	-
Age, y (Mean/SD)	(64.5/12.1)		(65.9/13.5)		(63.5/10.5)		(63.9/12.0)		0.396^b^
≤ 39	6	3.6	4	6.3	1	1.6	1	2.3	
40-49	10	5.9	2	3.1	5	8.2	3	6.8	
50-59	39	23.1	12	18.8	13	21.3	14	31.8	0.0524
60-69	50	29.6	19	29.7	20	32.8	11	25.0	
70-79	48	28.4	16	25.0	22	36.1	10	22.7	
≥ 80	16	9.5	11	17.2	0	0.0	5	11.4	
Missing information	6	-	3	-	2	-	1	-	-

Gender									

Female	64	36.6	34	50.7	13	20.6	17	37.8	0.0017
Male	111	63.4	33	49.3	50	79.4	28	62.2	

HPV									

Negative	142	81.1	51	76.1	52	82.5	39	86.7	0.3530
Positive	33	18.9	16	23.9	11	17.5	6	13.3	

a Chi-Square Test;

b Kruskal-Wallis test.

### HPV genotyping

Table 2 describes the prevalence of HPV and HPV associated factors among the 175 samples. The overall prevalence of HPV DNA in HNSCC cases was 18.9%. HPV was found in 23.9%, 17.5% and 13.3% of oral cavity, larynx and oropharynx cases respectively. Twenty-seven samples (15.4%) were positive for HPV 16 and 6 (3.42%) for HPV18. Although the mean age of the cases was around 64 years, we found a higher prevalence of HPV among younger cases. Indeed, the mean age/(SD) of HPV positive cases was 60.1/(10.14) years meanwhile the mean age/(SD) of the negative cases was 65.5/(12.3) years and this difference was statistically significant (Mann-Whitney test *p* = 0.02).

**Table 2:** Prevalence and characteristics associated with HPV infection in Head and Neck squamous cell carcinoma cases of clinical centers of Medellin, Colombia.

Characteristics	Positive^a^		Negative		OR (95% CI)	*p*	ORb (IC 95%)	pb
	n	(%)	n	(%)				
Total	33	(18.9)	142	(81.1)	-	-		
Age, y (Mean/SD)	(60.1/10.4)		(65.5/12.3)		-	0.02^c^		
≤ 39	2	(33.3)	4	(66.7)	1	-		
40-49	1	(10.0)	9	(90.0)	0.22 (0.02-3.22)			
50-59	11	(28.2)	28	(71.8)	0.79 (0.13-4.92)	0.005	-	-
60-69	14	(28.0)	36	(72.0)	0.78 (0.13-4.73)			
70-79	4	(8.3)	44	(91.7)	0.18 (0.03-1.32)			
≥ 80	0	(0.0)	16	(100)	0.00	-		
Missing values	1	-	5	-	-	-		

Gender								

Females	8	(12.5)	56	(87.5)	1	0.09	1	0.356
Males	25	(22.5)	86	(77.5)	2.03 (0.86-4.83)		1.53 (0.61-3.85)	

Anatomic site								

Oral cavity	16	(23.9)	51	(76.1)	1	0.350	1	0.153
Larynx	11	(17.5)	52	(82.5)	0.67 (0.29-1.59)		0.49 (0.19-1.24)	
Oropharynx	6	(13.3)	39	(86.7)	0.49 (0.18-1.37)		0.39 (0.13-1.16)	

a 27 HPV 16 and 6 HPV 18 positive;

b Adjusted by age;

c Mann-Whitney test.

The proportion of HPV positive HNSCC cases was higher in males (22.5%) than in females (12.5%) and the probability of HPV positivity of HNSCC cases was two times higher in males than females, although this higher probability was not statistically significant (OR 2.0, 95% CI: 0.86-4.83) and decreased after adjusting for age (OR: 1.53, 95% CI: 0.61-3.85). Although the proportion of HPV-positive cases was higher among cases of oral cavity, than in the cases of larynx and oropharynx, these differences were not statistically significant and remained insignificant after adjustment by age. The analysis restricted to cases positive for HPV 16, also showed that the mean age (60.3/11.4) of positive cases was lower than that of negative cases (65.3/12.1), but this difference did not reach statistical significance (Mann-Whitney test *p* = 0.06). There was not the difference between percentages of HPV positive HNSCC cases diagnosed in the period 1999-2003 and 2004-2008 (data not shown).

## DISCUSSION

The causal association between HPV and a subset of HNSCC, has been demonstrated^5^, ^6^, ^7^ and studies of case series suggest that HPV is associated with a proportion of about 25-60% of these cases^12^. The confirmation of HPV association with these cancers opens the opportunity for implementing prevention strategies based on the control of HPV infection. Currently available HPV prophylactic vaccines have shown a nearly 100% efficacy in preventing infection with HPV 16 and 18, with a projected reduction in risk of cervical cancer by 50-70% and significant impact on non-genital HPV related diseases^22^. The ability of these vaccines to reduce the incidence of these cancers may depend partly on the proportion of cases attributed to the genotypes included in current available vaccines. Estimates of the contribution of HPV genotypes in HNSCC are scarce in Latin American countries^12^.

In this article we describe the demographic characteristics of 175 cases of HNSCC consecutively diagnosed over a period of 10 years (1999-2008) and the prevalence of HPV in these cases. Our results show similar prevalence of HPV found in other studies conducted in several regions worldwide^12^ but higher than the prevalence described in a study including cases from Brazil, Cuba and Argentina^18^. These findings are consistent with observations on the geographical variability of the prevalence of HPV in HNSCC worldwide. Our study also found that among the positive HPV samples, HPV 16 was present in 82% of cases, while HPV 18 was observed only in 18% of cases. These results are consistent with widely reported data which implicates HPV 16 in most cases of HNSCC^12^, ^25^.

As it has been described^26^, we also noted that despite the high proportion of cases of HNSCC being diagnosed in people over 50 years, HPV-positive cases tend to be younger than HPV-negative (60.1 vs. 65.5, Mann-Whitney test *p* = 0.02), and this difference persisted even when the analysis included only cases positive for HPV 16 although this did not reach statistical significance (data not shown). The prevalence of HPV in HNSCC in this study is higher than that observed in cases from Brazil, Argentine and Cuba^18^. Due to the nature of our study, it was not possible to collect information on alcohol and tobacco use. It has been suggested that the proportions of anatomical sites included in the analysis can influence the observed prevalence differences.

The proportion of cancers of the oral cavity, larynx and oropharynx in that study was 31%, 49% and 19% respectively, which is similar to the proportion of cases included in our study. HNSCC is a malignant epithelial tumor with squamous differentiation characterized by the formation of keratin or the presence of intercellular bridges or both. Histopathologic characteristics that could be evaluated in the study biopsies and resections, demonstrated well to moderate differentiated SCC, mostly keratinizing tumors, moderate to severe peritumoral inflammatory infiltrate and expansive or infiltrative pattern of invasion regardless of anatomic sites. HPV has been associated as a risk factor for non-keratinizing HNSCC occurring in the tonsillar (oropharyngeal and oral) and sinunasal areas.

In our study, the proportion of oral cavity HPV-positive cases was higher than the proportion of larynx and oropharynx cases (sinunasal area not included). But these differences were not statistically significant and remained insignificant after adjustment by age. The lymphoid tissue of the tonsillar ring in the head and neck consists of the palatine, the pharyngeal and the lingual tonsils, which means it is distributed mainly along the oropharynx but the oral cavity too. It is worth noting that among the evaluated biopsies many were incisional biopsies that represent mainly the superficial area of the tumor and did not include representation of the non-neoplasic tissues, where tonsillar tissue may be recognized more easily.

Even though tonsillar tissue was not observed in these oral cavity biopsies, we cannot exclude from the histological slides that in some cases the tumor could involve or originated from tonsils and that this could explain the higher prevalence of HPV DNA in cases of oral cavity. In the same direction, maybe our oropharynx cases did not include as many tumors from the tonsils and thus the HPV prevalence may have been underestimated. Even further, it has been reported that recognition of non-keratinizing morphology has low reproducibility^27^. Another reason may be the methods used to determine exposure to HPV. For the assessment of prevalence estimates in HNSCC, serological tests that detect antibodies against L1 protein, E6 and E7 have been used.

Although these essays are useful biomarkers to define the role of HPV in HNSCC they may have some limitations to estimate the fraction of these cancers attributable to HPV. For example, antibodies against L1 are markers of current or previous exposure to HPV infection at any anatomical site, including anogenital infections and its correlation with the presence of DNA at the site of the lesion is very poor^28^. Antibodies against the E6 and E7 proteins correlate with invasive cancer at least in cervical cancer, but only 60%-70% of HNSCC cases are positive for these antibodies^29^. In cervical cancer, the most accurate method in the assessment of the exposure to HPV has been PCR of HPV DNA extracted from tumor microdissected lesions^30^, but the use of paraffin blocks requires the implementation of proven methods with high sensitivity in paraffin-embedded material.

Although fresh frozen tumor biopsies produce good quality DNA, they have the limitation that identification and microdisection of the tumor tissue cannot be conducted. Indeed, strength of our study is the use of paraffin blocks, which were re-cut for central pathology review and extraction of DNA from the dissected material of tumors identified by the study pathologist. We also evaluated the quality of DNA by amplifying a segment of 209 bp of the β-globin gene and use 2 type specific PCR tests that amplify small size fragments (96 pb for HPV 16 and 115 pb for HPV 18) of the E6 gene which have shown to be highly sensitive in this type of sample^24^.

Although we cannot completely exclude the possibility of false negatives due to technical artifacts, the use of highly sensitive type-specific PCR for HPV 16 and 18 may help to overcome this limitation, especially since these two are the predominant genotypes in HNSCC. Potential limitations of our study include possible selection bias of samples. The cases were collected from only four local laboratories, but we cannot exclude the likelihood that these cases do not represent the entire population of cases of HNSCC diagnosed in the geographic area. Compared with Brazil, Cuba and Argentina, the incidence rate of HNSCC in Colombia is lower^31^. Likewise, cigarette smoking and annual cigarette consumption per person is lower in Colombia^32^, ^33^. It would be useful to evaluate HPV prevalence in HNSCC cases in the context of these epidemiological profiles to ascertain the fraction attributed to each risk factor in HNSCC.

## CONCLUSION

The HPV prevalence in these series of cases of four laboratories of the city of Medellin, Colombia was 18.9%. HPV was found in 23.9%, 17.5% and 13.3% of oral cavity, larynx and oropharynx cases respectively. Twenty-seven and six samples were HPV 16 (15.4%) and HPV 18 (3.42%) respectively. Among HPV positive cases, 82% were HPV 16 and 18% HPV 18. No other HPV genotypes were identified. According to these data, the mass introduction of prophylactic HPV vaccines may have the potential to reduce the number of HNSCC cases in Colombia, but the magnitude of this effect is difficult to predict at this time because clinical trials which are specifically designed to evaluate the efficacy of these vaccines in the prevention of HPV oral infection and on cancers of the head and neck have not conducted.
